# A controlled evaluation of filter paper use during staining of sputum smears for tuberculosis microscopy

**DOI:** 10.12688/wellcomeopenres.18827.1

**Published:** 2023-04-14

**Authors:** Nataly Bailon, Eric Ramos, Keren Alvarado, Lenin Bernaola, James Wilson, Rosario Montoya, Teresa Valencia, Carlton A Evans, Sumona Datta

**Affiliations:** 1 IFHAD: Innovation For Health And Development, Universidad Peruana Cayetano Heredia LID 416, Lima, Peru; 2 IPSYD: Innovacion Por la Salud Y el Desarollo, Asociación Benéfica Prisma, Lima, Peru; 3 IFHAD: Innovation For Health And Development, Department of Infectious Disease, Imperial College London, London, W12 0NN, UK; 4 Department of Clinical Sciences, Liverpool School of Tropical Medicine, Liverpool, UK

**Keywords:** Filter paper, tuberculosis, Ziehl Neelsen, Auramine, fluorescein diacetate, sputum smear, acid-fast bacilli, microscopy

## Abstract

**Background**: Some sputum smear microscopy protocols recommend placing filter paper over sputum smears during staining for *Mycobacterium tuberculosis *(TB) *. *We found no published evidence assessing whether this is beneficial. We aimed to evaluate the effect of filter paper on sputum smear microscopy results.

**Methods:** Sputum samples were collected from 30 patients with confirmed pulmonary TB and 4 healthy control participants. From each sputum sample, six smears (204 smears in total) were prepared for staining with Ziehl-Neelsen (ZN), auramine or viability staining with fluorescein diacetate (FDA). Half of the slides subjected to each staining protocol were randomly selected to have Whatman grade 3 filter paper placed over the dried smears prior to stain application and removed prior to stain washing. The counts of acid-fast bacilli (AFB) and precipitates per 100 high-power microscopy fields of view, and the proportion of smear that appeared to have been washed away were recorded. Statistical analysis used a linear regression model adjusted by staining technique with a random effects term to correct for between-sample variability.

**Results:** The inclusion of filter paper in the staining protocol significantly decreased microscopy positivity independent of staining with ZN, auramine or FDA (p=0.01). Consistent with this finding, there were lower smear grades in slides stained using filter paper versus without (p=0.04), and filter paper use reduced AFB counts by 0.28 logarithms (95% confidence intervals, CI=0.018, 0.54, p=0.04) independent of staining technique. In all analyses, auramine was consistently more sensitive with higher AFB counts versus ZN (p=0.001), whereas FDA had lower sensitivity and lower AFB counts (p<0.0001). Filter paper use was not associated with the presence of any precipitate (p=0.5) or the probability of any smear washing away (p=0.6) during the staining process.

**Conclusions:** Filter paper reduced the sensitivity of AFB microscopy and had no detectable beneficial effects so is not recommended.

## Introduction

Tuberculosis (TB) is an infectious disease caused by *Mycobacterium tuberculosis*, which in 2019 caused the death of 1.2 million people worldwide ^1^. Although there are more sensitive laboratory methods for diagnosing TB, such as culture and PCR, sputum smear microscopy remains the most widely used method due to its low cost, minimal required infrastructure, high specificity and rapidity ^2– 5^.

Since the discovery of TB bacilli in 1882 by Koch’s staining and microscopy method, other mycobacteriologists including Ehrlich, Rindfleisch, Ziehl, and Neelsen, optimized these protocols until recommending the widely used Ziehl-Neelsen (ZN) ^6^ protocol using carbol fuchsin as the primary stain with an acid-alcohol wash. ZN is the most used staining technique for TB smear microscopy due to its simplicity, robustness and cost-effectiveness ^5^. In 2009, the World Health Organization recommended that TB smear microscopy should be carried out using auramine staining and fluorescent microscopy due to higher sensitivity (compared to ZN staining with normal light microscopy) ^7^ and the increasing availability of inexpensive fluorescent microscopes using light-emitting diodes (LED). Meanwhile, there has been growing interest in the use of fluorescein diacetate (FDA) which unlike conventional TB microscopy with ZN or auramine, is a viability stain that stains live but not dead bacilli ^8^. Therefore, FDA may be potentially useful in monitoring response to TB treatment ^9^.

During the development of all three aforementioned staining methods for TB microscopy (ZN, auramine and FDA), the application of filter paper during staining has been recommended because it was thought to improve staining and some TB diagnostic laboratories still use filter paper in their protocols, placing it on top of the sputum smear before adding the primary stain ^8, 10– 13^. Filter paper use during staining could increase fixation of the dyes to the smear, in the same way as when water ‘wets’ human skin differently when covered by a porous material such as cotton cloth. Applying stains to filter paper resting on the sputum smear may also reduce the potentially confusing appearance of stain precipitates, which can be mistaken for atypical acid-fast bacilli. However, we could find no published literature assessing the effects of filter paper on these protocols and no evidence demonstrating benefit. Therefore, the aim of this study was to evaluate the impact of filter paper use during staining on microscopy sensitivity and slide quality.

## Methods

### Ethics

Internationally accredited ethics committees approved the study, including Imperial College London, UK (reference 14IC2191), Asociacion Benefica PRISMA, Peru (reference CE0970.16), and the Peruvian Ministry of Health DIRESA Callao (reference 790-2014-DG). This research was done with the collaboration of the Peruvian national TB program. All patients gave informed written consent.

### Study participants

“Spot” sputum samples were collected from 30 adult (≥18 years old) patients starting or receiving treatment for pulmonary TB from community health posts in Callao, Peru ^2^. This recruitment of participants constituted part of a larger cohort study (ISRCTN, https://doi.org/10.1186/ISRCTN17820976)). We collected these samples from 17/09/2018 until 03/11/2018. Participants were selected consecutively on the basis of recent programmatic sputum smear microscopy ZN results in order to provide approximately equal numbers of patient samples with each sputum smear microscopy grade (negative, paucibacillary, +, ++, and +++) ^14^. All patient samples had the presence of *M. tuberculosis* confirmed either by PCR testing with the GeneXpert MTB/RIF test or culture using the thin-layer agar Colour Test technique ^15^. “Spot” sputum was also collected from four healthy, asymptomatic control participants.

### Sample processing

**Figure 1.**
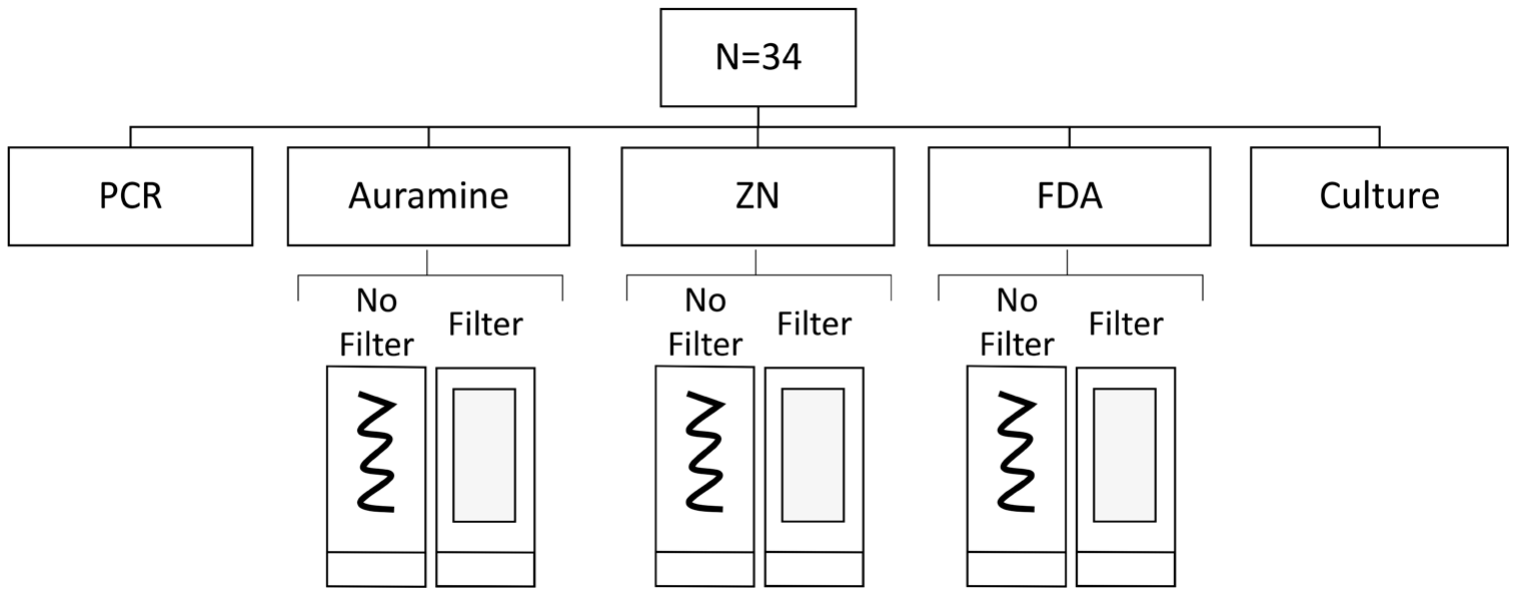
Diagnostic tests performed including 204 microscopy slides from all 34 samples included in the study. PCR and culture testing were omitted for the 4 samples from healthy control participants. Note: ZN indicates Ziehl-Neelsen and FDA indicates fluorescein diacetate.

The overview of the study is shown in Figure 1. Prior to smear preparation, standard glass slides were cleaned with 95% alcohol, air dried, and then an area of 3 cm by 1 cm were marked on the underside with indelible ink, to indicate the standardised area to apply the smear. Six smears were made from each sputum sample, by adding a drop of sputum using a disposable transfer pipette (approximately 40 µL of sputum) and spreading it over the indicated area. Smears were then dried on a slide warmer for 1 hour at 40°C and passed through a Bunsen burner flame for heat fixation.

### Filter paper

**Figure 2.**
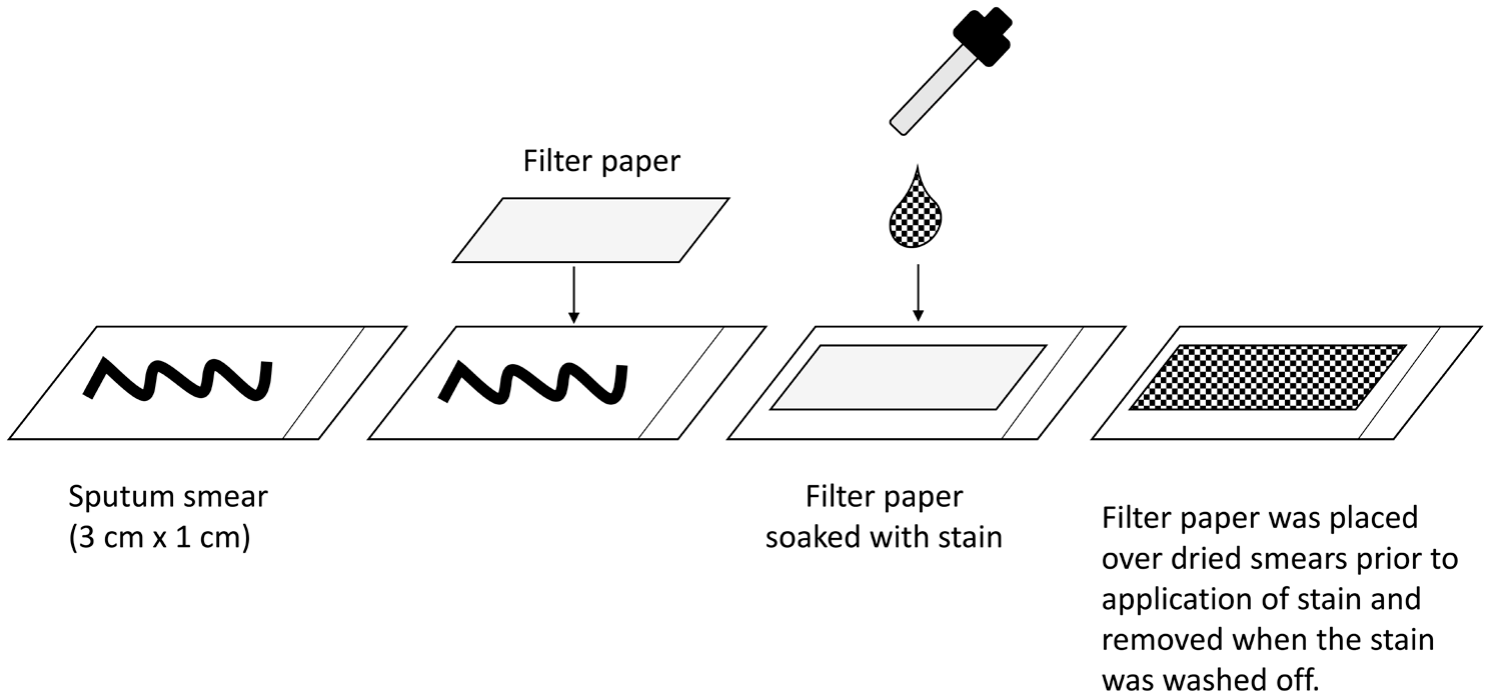
Diagram of how filter paper was used to stain each smear with Ziehl-Neelsen (ZN), auramine (AR) and fluorescein diacetate (FDA).

For each sample, alternate slides selected in random order had a 3 cm by 1 cm piece of Whatman grade 3 filter paper placed over the dried smear ( Figure 2).

### Ziehl-Neelsen staining

Two smears from each sample, one with and one without filter paper, were flooded with 0.3% basic carbol fuchsin. The underside of the slide was then heated with a Bunsen burner until a vapour was realised from the stain without boiling. This heating step was repeated three times. After five minutes, the filter paper was discarded with tweezers from the half of slides to which filter paper had been applied. Then, all the slides were washed with distilled water. The smears were then decolorized by flooding the slide with 3% hydrochloric acid-alcohol solution for two minutes, and then washed again with distilled water. The slide was then flooded with 0.3% methylene blue for two minutes to add contrast to the smears, washed with distilled water and left to dry in a dark place.

### Auramine staining

Auramine at a concentration of 0.1% was flooded over smears with or without filter paper and left for 15 minutes. Using tweezers, the filter paper was removed and discarded from the half of slides to which filter paper had been applied. Then, all the slides were washed with distilled water. The smear was then decolorized with 0.5% hydrochloric acid-alcohol for two minutes and washed with distilled water. The smears were then flooded with 0.5% potassium permanganate for two minutes, to quench the background fluorescence, and then washed with distilled water. Slides were then left to dry in a dark place. 

### Fluorescein diacetate staining

FDA working solution at a concentration of 20 µg/mL was prepared beforehand as per the standard protocol ^3^. This solution was flooded over the smears with and without filter paper. The slides were then incubated at 37°C for 30 minutes. Using tweezers, the filter paper was removed and discarded from the half of slides to which filter paper had been applied. Then, all the slides were washed with distilled water. The slides were then flooded with 0.5% hydrochloric acid-alcohol for three minutes, and then washed with distilled water. The smears were then disinfected with 5% phenol for 10 minutes, washed with distilled water and then quenched with 0.5% potassium permanganate for 30 seconds. After the final wash with distilled water, the slides were left to dry in a dark place. 

### Microscopy

Once slides were dry, they were recoded so that the microscopists were blinded to which slides had been stained with or without filter paper. All slides were read within 24 hours with the Zeiss Primo Star (Heidenheim, Germany) iLED microscope using the 10× eyepiece and 100× objective with oil immersion. Slides stained with ZN were read with normal white light, whereas other slides (stained with auramine and FDA) were read with the blue 455 nm LED light source. The number of acid-fast bacilli (AFB) visualized in 100 consecutive adjacent microscopy fields of view was counted and recorded for each slide. From now on, this count is referred to as AFB counts per 100 fields. The effect of filter paper on artefacts was also recorded, where artefacts in microscopy were defined as precipitates of the smear washing away. Prior to study commencement, the investigators and microscopists had all agreed on what would be considered as a precipitate *versus* bacilli. Subsequently, the number of precipitates were also recorded per 100 microscopy fields for each slide. Furthermore, before reading each slide, microscopists recorded an estimation of the percentage of smear that appeared to have washed away during the staining process, if any. Both precipitate counts and percentage smear washing away were considered as *a priori* explanatory variables, that could contribute to the benefit or disadvantage of filter paper use.

### Statistical analysis

Microscopy readings with ≥1 AFB count were considered positive, and smear grades were calculated from the AFB count per 100 microscopy fields as: paucibacillary=1-9 AFB; “+”=10-99 AFB; “++”=100-999 AFB; and “+++”≥1000 AFB. AFB counts were transformed to the logarithm base 10 (log10) values and analysed as a continuous variable. Prior to logarithmic transformation, zero values were replaced by a value of 0.3 (the midpoint between zero and the detection threshold). All statistical analyses were performed with Stata version 17 (College Station, TX: StataCorp LP). As shown in Figure 1, all samples were tested in parallel using all three staining techniques with and without the presence of filter paper during staining. Consequently, to be able to assess the overall effect of filter paper on microscopy (AFB counts, precipitate/artefact counts and percentage smear washing), we used a linear regression model adjusting for the staining technique with a random effects term to correct for between-sample variability.

## Results

### Negative controls

Patients and their sample characteristics are shown in Table 1. All smears from healthy controls had entirely negative microscopy results ( Table 2) and are not included in any analyses.

**Table 1.** Patient and patient sample characteristics (N=30). Note: IQR=inter-quartile range; SD=standard deviation; and N=number.

Variables	Results
Age, median (IQR)	28 (23-39)
Male, % (n)	67 (20)
Rifampicin resistant tuberculosis, % (n)	6.7 (2)
Previous TB treatment, % (n)	20 (6)
Treatment duration prior to sample collection, median days (IQR)	0 (0-1)
Delay in processing, median days (IQR)	1 (0-3.3)
Sputum sample volume, mean (SD)	6.1 (1.7)
Sputum macroscopic consistency, % (n)	
Salivary, watery	53 (16)
Muco-purulent	47 (14)
Semi-solid	0 (0)

**Table 2.** Summary of microscopy results in patient samples. Note: log10=logarithm base 10 values, ZN=Ziehl Neelsen, AFB=acid fast bacilli; FDA=fluorescein diacetate, IQR = inter-quartile rang, SD= standard deviation, and N=number.

			Auramine	ZN	FDA
Positivity in controls (n=4)	% (n/N)	no filter	0% (0/4)	0% (0/4)	0% (0/4)
		filter	0% (0/4)	0% (0/4)	0% (0/4)
Patient samples (n=30):					
Positivity	% (n/N)	no filter	87% (26/30)	90% (27/30)	63% (19/30)
		filter	83% (25/30)	80% (24/30)	60% (18/30)
Smear grade	% + or++ or +++ (n/N)	no filter	80% (24/30)	73% (22/30)	46% (14/30)
		filter	77% (23/30)	57% (17/30)	30% (9/30)
Mean AFB count/100 fields	log10 (SD)	no filter	2.0 (1.2)	1.6 (1.2)	0.75 (1.2)
		filter	1.7 (1.4)	1.5 (1.4)	0.56 (1.1)
Median number of precipitates	count (IQR)	no filter	0 (0-1.0)	0 (0-1.3)	0 (0-0)
		filter	0 (0-2.3)	0 (0-2.0)	0 (0-0)
Median percentage washed off	% (IQR)	no filter	0% (0-0)	0% (0-0)	20% (0-70)
		filter	0% (0-0)	0% (0-0)	20% (0-73)

### Patient characteristics

The patients had a median age of 28 (interquartile range (IQR)=23-39) years and 67% (n/N=20/30) were male. 80% (n/N=24/30) of samples were collected prior to treatment initiation, and 77% (n/N=23/30) of samples were processed within three days of sputum collection.

### Positivity

**Figure 3.**
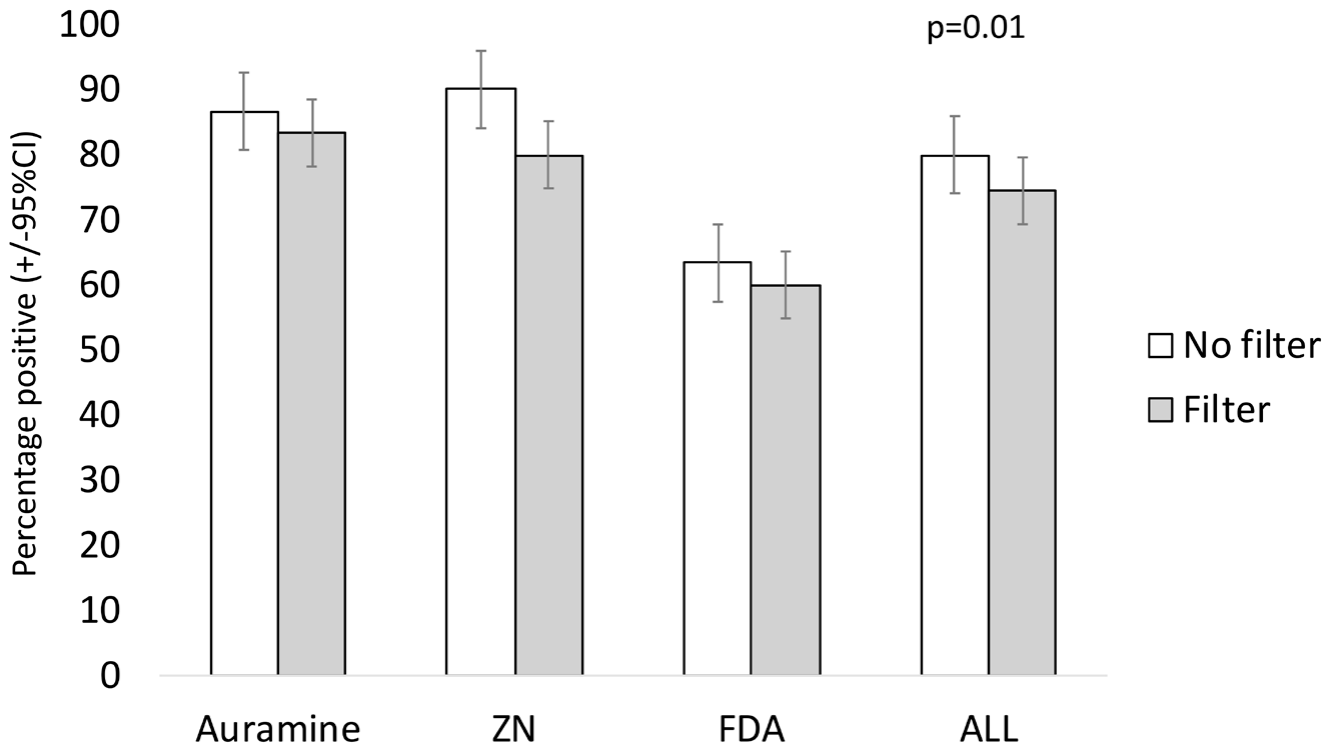
Percentage positivity of slides stained without versus with filter paper used during staining. Error bars indicate 95% confidence intervals (CI). The P value is from a linear regression model with a random effects term shown in Table 3, whereas crude data are shown graphically. Note: ZN indicates Ziehl-Neelsen and FDA indicates fluorescein diacetate.

In patient samples, 85% (n/N=51/60) of auramine stained slides, 85% (n=51/60) of ZN-stained slides, and 62% (n/N=37/60) of FDA-stained slides were positive. Figure 3 and Table 3 show that filter paper use significantly decreased the positivity of microscopy independent of staining technique (odds ratio (OR)=0.25, 95% confidence interval (95% CI) = 0.085, 0.74 p=0.01).

**Table 3.** Multivariable regression of microscopy sensitivity. Sensitivity is presented as (a) positivity; (b) smear grade; and (c) AFB counts adjusted by staining type. All generalised linear regression models were analysed with a random effects term, as described in the Methods. Note: vs=versus; CI=confidence intervals; ZN=Ziehl-Neelsen; and FDA=fluorescein diacetate.

	(a) Positivity Logistic regression Odds ratio (95%CI) p value	(b) Smear Grade Ordinal regression Odds ratio (95%CI) p value	(c) AFB count Negative binomial regression Coefficient (95%CI) p value
**Filter** (vs no filter)	0.25 (0.085, 0.74) p=0.01	0.52 (0.028, 0.96) p=0.04	-0.28 (-0.54, -0.018) p=0.04
**Auramine** (vs ZN)	5.5 (1.3, 23) p=0.02	2.0 (0.98, 4.2) p=0.06	0.49 (0.020, 0.78) p=0.001
**FDA** (vs ZN)	0.073 (0.018, 0.029) p<0.0001	0.068 (0.028, 0.17) p<0.0001	-1.3 (-1.7, -0.92) p<0.0001

### Smear grades

**Figure 4.**
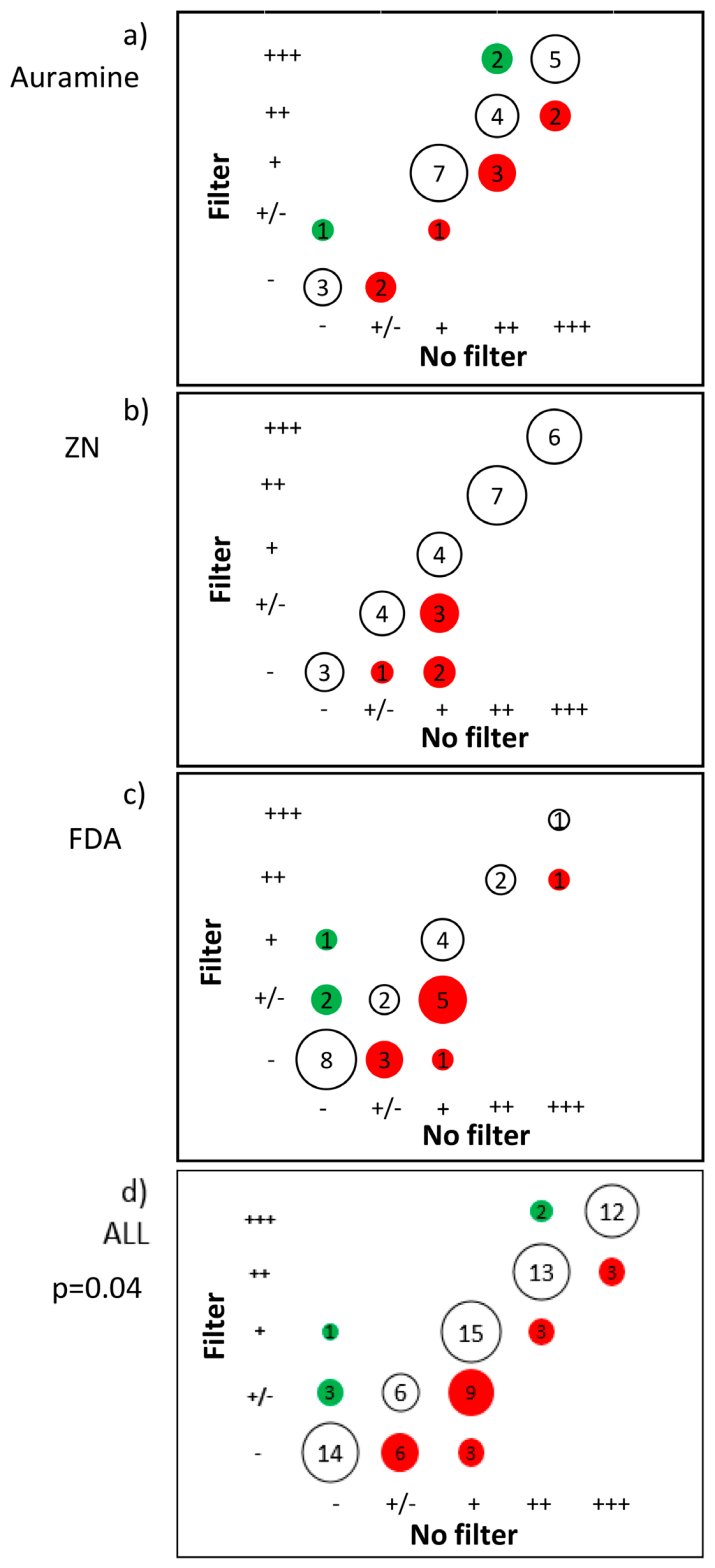
Matrix bubble plot comparing the smear microscopy grade without versus with filter paper used during staining. The numbers indicate the number of samples, which is proportional to the area of each circle (bubble). Note per 100 high-power fields visualized: - indicates negative; +/- indicates 1-10 bacilli; + indicates 10-99 bacilli; ++ indicates 100-999 bacilli; +++ indicates =>1000 bacilli; red circles indicate smear grade reduced with filter paper; and green circles indicate smear grade increased with filter paper. The P value is from a linear regression model with a random effects term shown in Table 3, whereas crude data are presented in all plots.

Consistent with the above finding, Figure 4 shows that smears stained using filter paper had lower smear grade results versus smears stained without filter paper. As shown in Table 3, filter paper use approximately halved the odds of having a higher smear grade independent of staining technique (OR=0.52, 95% CI) = 0.028, 0.96 p=0.04).

### AFB counts

**Figure 5.**
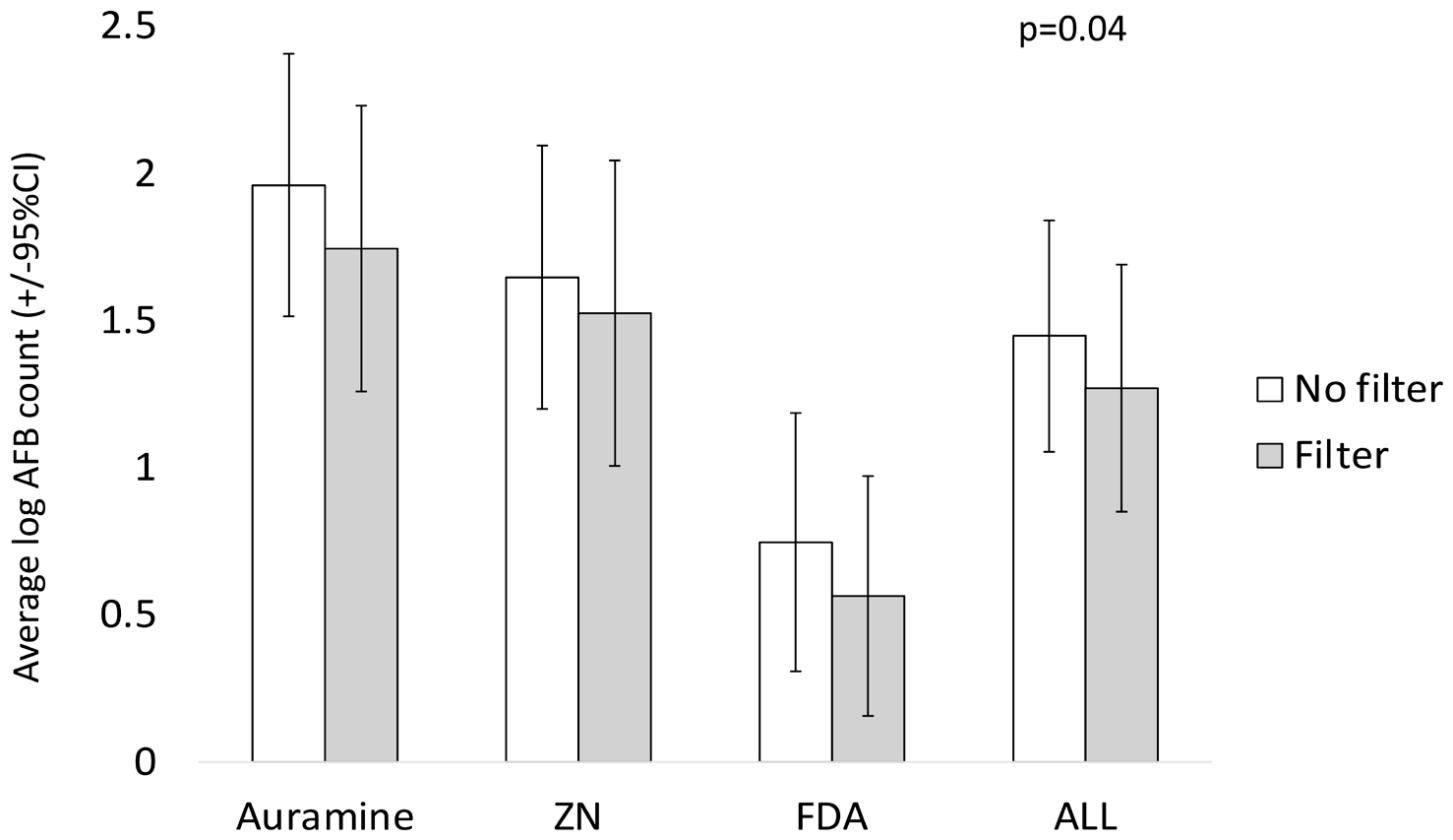
Average base-10 logarithmic acid-fast bacilli (log AFB) counts per 100 high-power fields visualized without versus with filter paper used during staining, Error bars indicate 95% confidence intervals (CI). The P value is from a linear regression model with a random effects term shown in Table 3, whereas crude data are shown graphically. Note: ZN indicates Ziehl-Neelsen and FDA indicates fluorescein diacetate.

Consistent with the results above, Figure 5 shows that filter paper use was associated with reduced mean AFB counts. In the regression model shown in Table 3, filter paper reduced the AFB count by 0.28 log (95% CI= 0.018, 0.54, p=0.04) independent of staining technique. Also, auramine was more sensitive with higher AFB counts versus ZN (p=0.001) and FDA had the lowest sensitivity with lower AFB counts (p<0.0001).

### Precipitates

**Figure 6.**
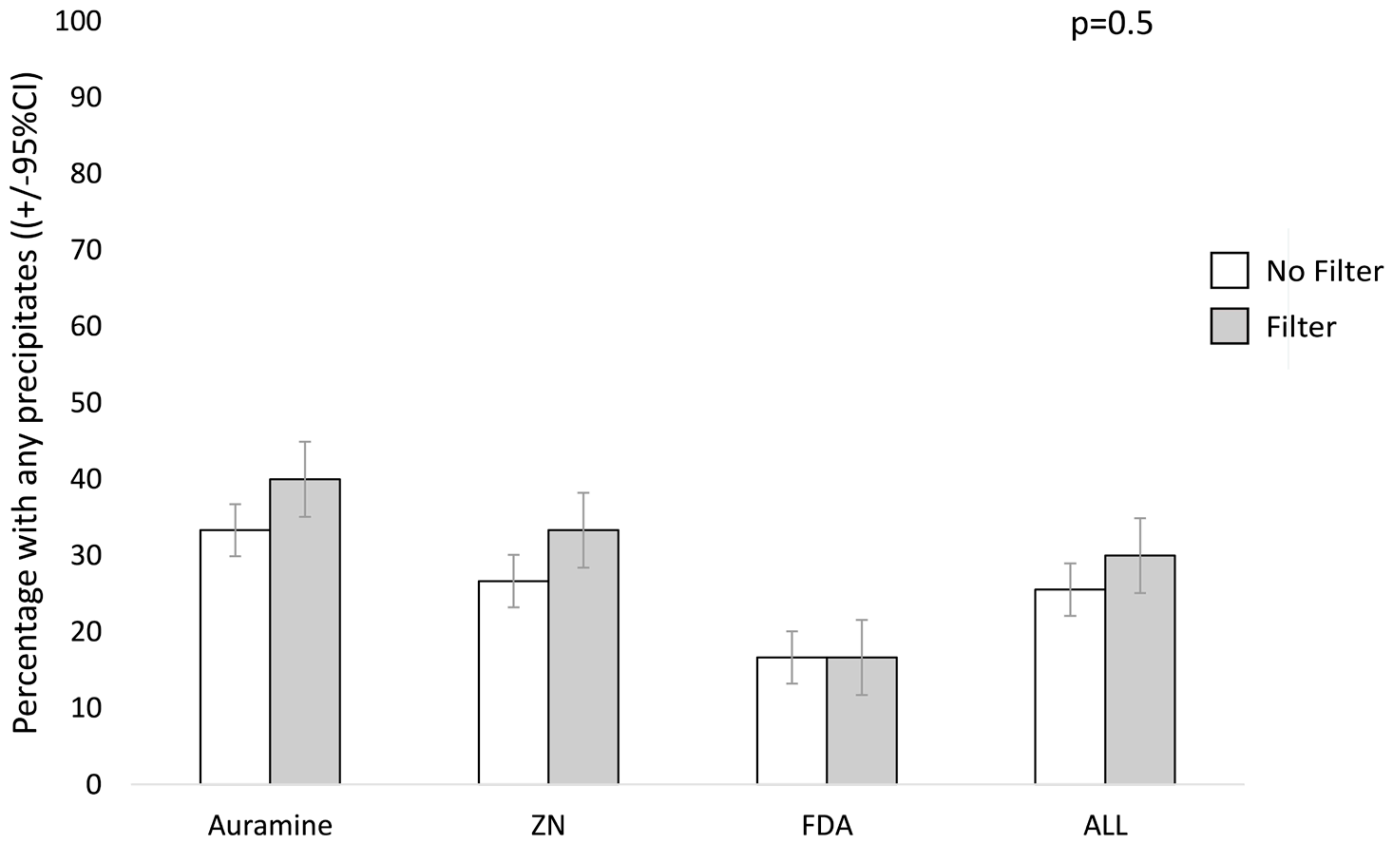
Percentage of slides with any precipitates visible per 100 high-power fields on each sputum smear without versus with filter paper. Error bars indicate 95% confidence intervals (CI). The P value is from a linear regression model with a random effects term shown in Table 4, whereas crude data are shown graphically. Note: ZN indicates Ziehl-Neelsen and FDA indicates fluorescein diacetate.

**Figure 7.**
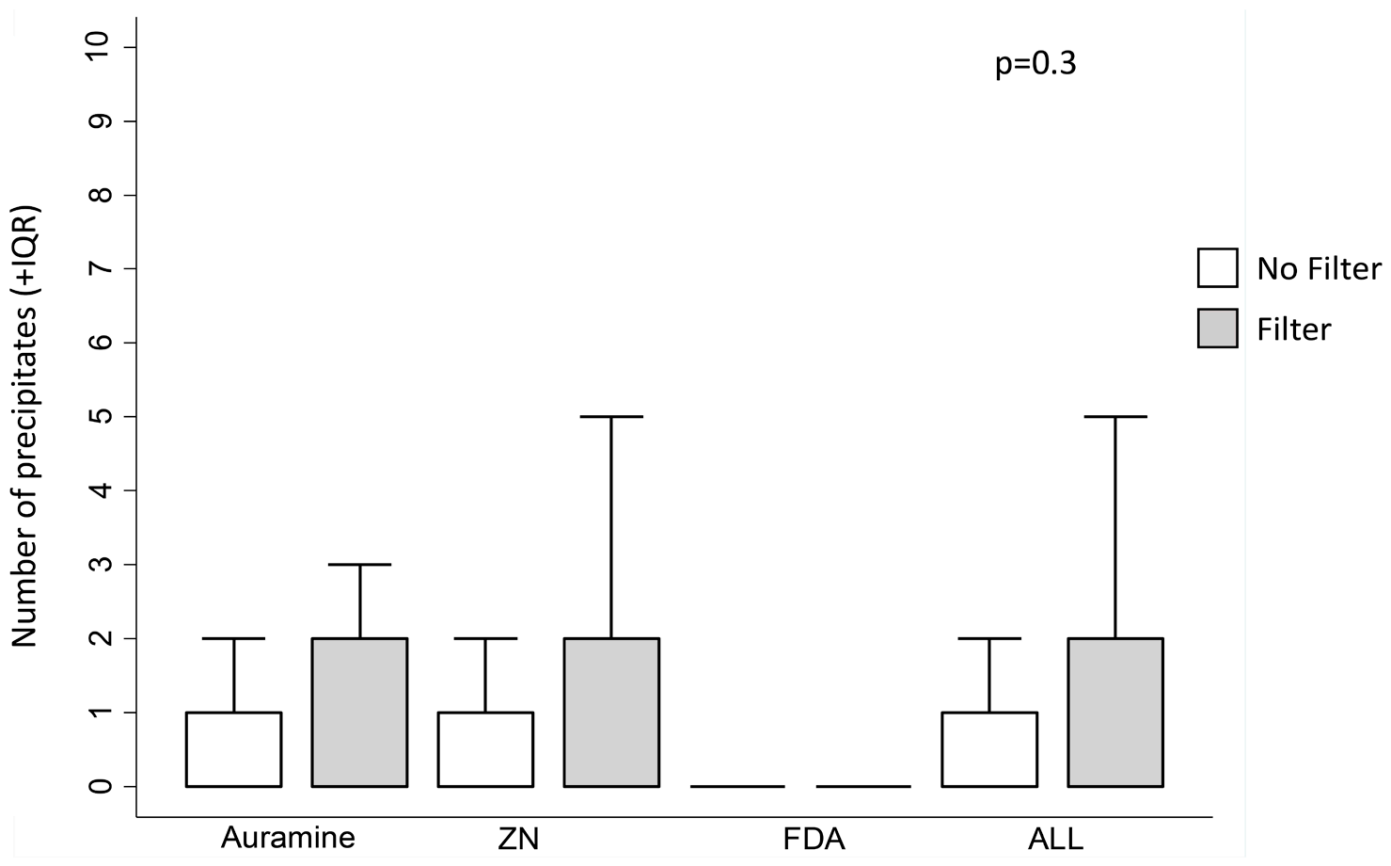
Box plot of the number of precipitates visible per 100 high-power fields on each sputum smear stained without versus with filter paper. The median value is shown by the horizontal line within the box, and outer box limits indicating the inter-quartile range (IQR). The P value is from a linear regression model with a random effects term shown in Table 4, whereas crude data are shown graphically. Note: ZN indicates Ziehl-Neelsen and FDA indicates fluorescein diacetate.

Crude analysis presented in Figure 6 and Figure 7 appeared to suggest a possible trend for filter paper increasing the probability of any precipitates being seen in the smear, and the number of precipitates in the smear, respectively. However, in the regression analyses, filter paper use was not significantly associated with either increased presence of any precipitate (p=0.5) or the number of precipitates (p=0.3), see Table 4. Smears stained with FDA tended to have less slides with any precipitate versus ZN (p=0.07, Table 4) and also versus auramine stained smears (OR=0.28, 95% CI=0.11, 0.73, p=0.009).

**Table 4.** Multivariable regression of artefacts in microscopy. Artefacts are presented as: (a-1) the presence of any precipitates; (a-2) the number of precipitates; (b-1) if any of the smear of washed off; and (b-2) the percentage of the smear washed off, all adjusted by staining type. All generalised linear regression models were analysed with a random effects term, as described in the Methods. Note. CI= confidence intervals, ZN=Ziehl-Neelsen and FDA=fluorescein diacetate.

	(a-1) Any precipitate Logistic regression Odds ratio (95%CI) p value	(a-2) Number of precipitates Negative binomial regression Coefficient (95%CI) p value	(b-1) Any washed away Logistic regression Odds ratio (95%CI) p value	(b-2) Percentage washed away Negative binomial regression Coefficient (95%CI) p value
**Filter** (Vs no filter)	1.3 (0.64, 2.7) p=0.5	0.29 (-0.26, 0.84) p=0.3	0.80 (0.32, 2.0) p=0.6	-0.11 (-0.68, 0.45) p=0.7
**Auramine** (Vs ZN)	1.4 (0.62, 3,3) p=0.4	0.16 (-0.45, 0.78) p=0.6	0.43 (0.094, 2.0) p=0.3	-0.74 (-2.1, 0.64) p=0.3
**FDA** (Vs ZN)	0.40 (0.15, 1.1) p=0.07	-0.72 (-1.4, 0.04) p=0.07	30 (7.6, 115) p<0.0001	2.4 (1.5, 3.3) p<0.0001

### Percentage of smear that washed away

**Figure 8.**
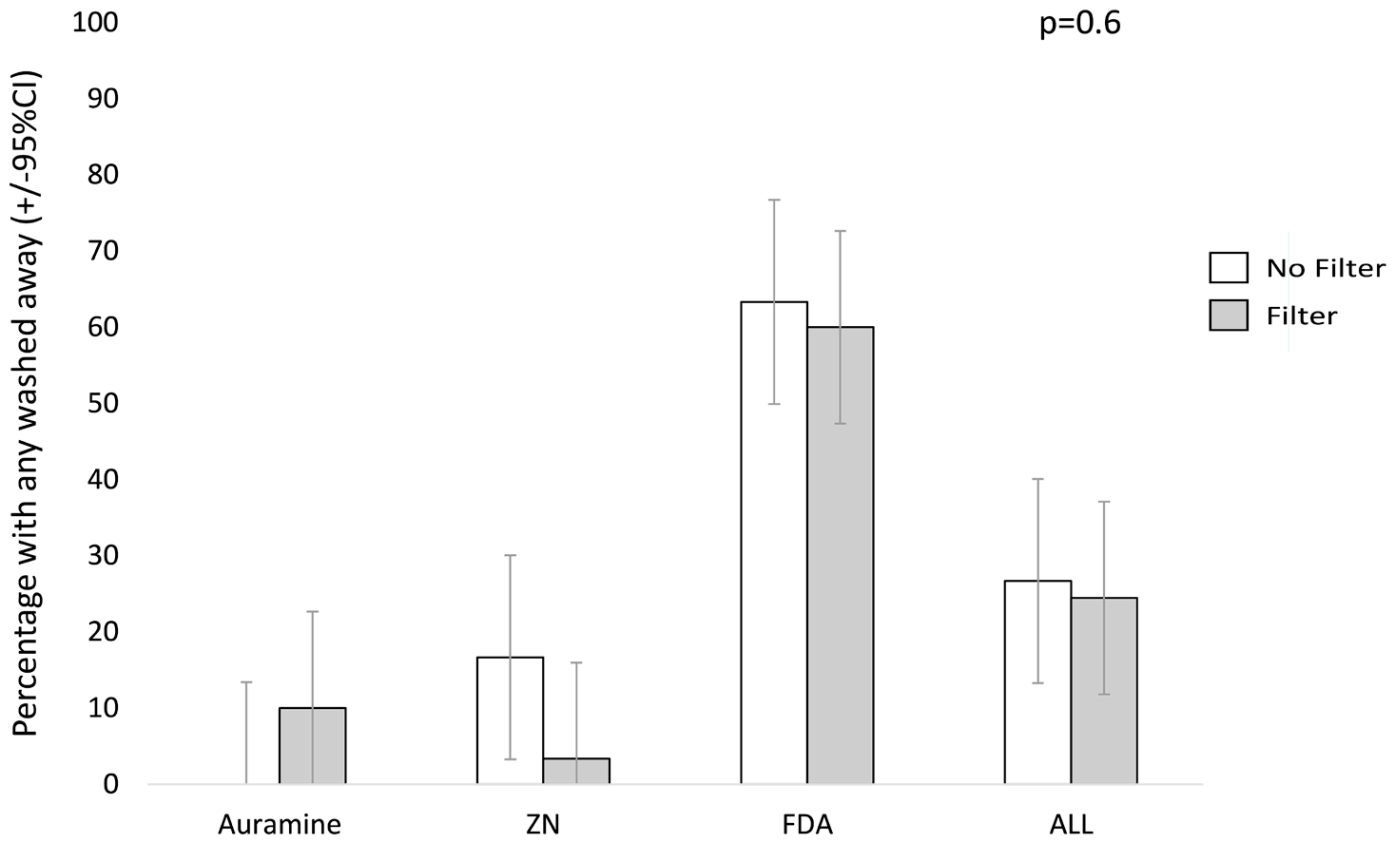
Percentage with any of the sputum smear area that appeared to have been washed away during staining on each sputum smear without versus with filter paper. Error bars indicate 95% confidence intervals (CI). The P value is from a linear regression model with a random effects term shown in Table 4, whereas crude data are shown graphically. Note: ZN indicates Ziehl-Neelsen and FDA indicates fluorescein diacetate.

**Figure 9.**
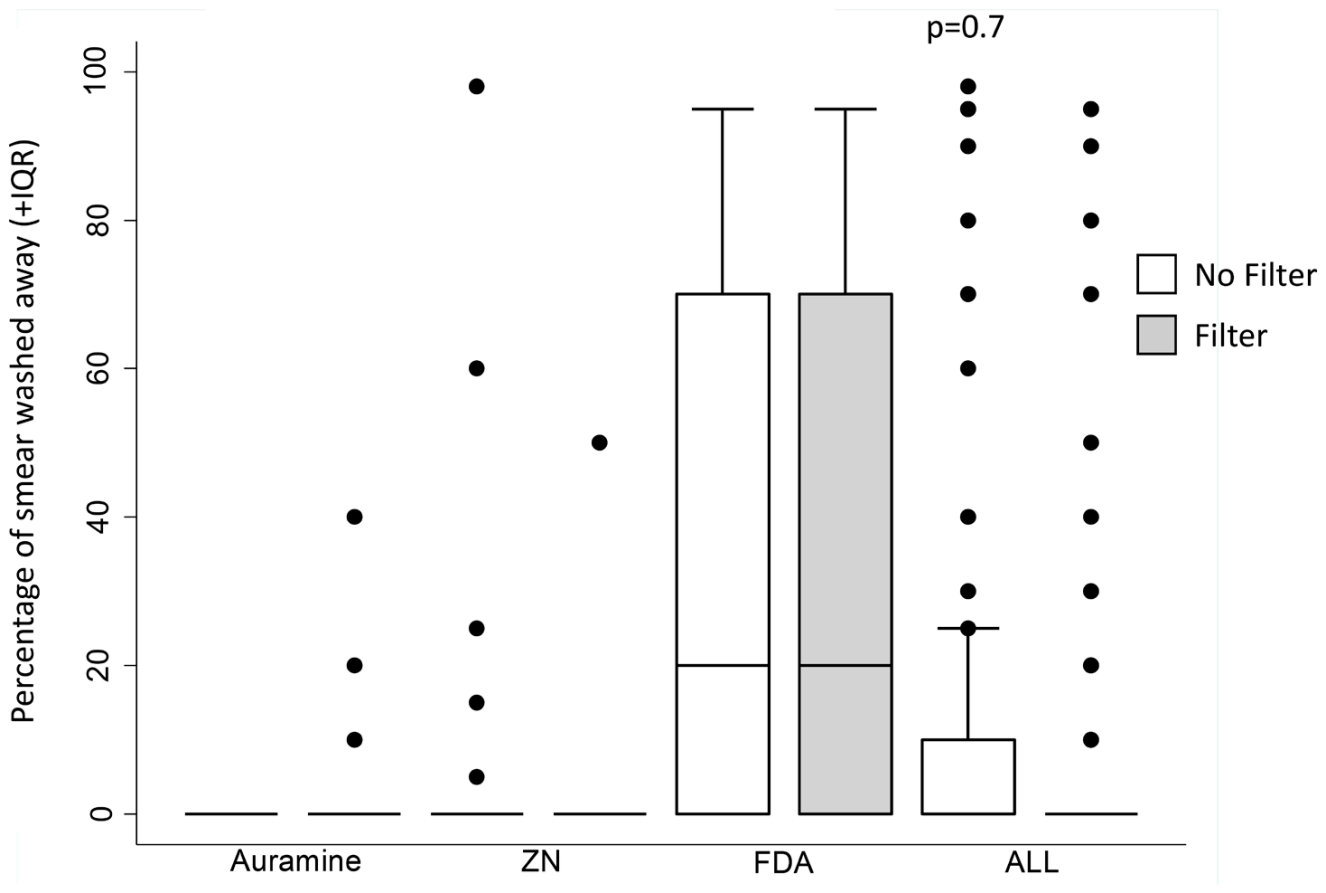
Box and whisker plots of the percentage of the sputum smear area that was estimated to have been washed away during staining. The median value is shown by the horizontal line within the box, and outer box limits indicating the inter-quartile range (IQR). Outlier data are shown as individual points. The P value is from generalised linear regression model with a random effects term shown in Table 4, whereas crude data are shown graphically. Note: ZN indicates Ziehl-Neelsen and FDA indicates fluorescein diacetate.

Approximately a quarter of all patient slides (26%, n/N=46/180) appeared to have partial washing away of smears during the staining process. FDA-stained smears had the greatest number of slides that appeared to have some of the smear washed away during the staining process (62%, n/N=37/60). Crude analysis presented in Figure 8 and Figure 9 appeared to suggest that filter paper use may tend to decrease the smears washing away during the staining with ZN or FDA, whilst increased smear washing away in auramine. However, in the regression analysis ( Table 4), filter paper was not associated with the probability of any smear washing away or the percentage washed away during the staining process (p=0.6 and p=0.7 respectively) independent of staining technique. However, FDA was consistently associated with a higher probability of any part of the smear washing away (p<0.0001) and higher percentage of smear washed washing versus ZN (p<0.0001).

## Discussion

This study provides evidence that the use of filter paper in sputum smear microscopy for TB testing had no detectable beneficial role and significantly reduced microscopy sensitivity. We therefore recommend that laboratories that routinely using filter paper in their smear microscopy protocols stop doing so.

Sputum microscopy plays a central role in TB diagnostics, especially in areas with limited resources ^4^. Conventional sputum smear microscopy has low sensitivity, but this is highly variable depending on multiple factors such as: sample viscosity; mycobacterial load; and operator experience ^16^. Therefore, strategies that improve this core laboratory technique will strengthen laboratory services in low resource settings and have the potential to positively impact patient care ^17^. We could not find any published literature regarding the effects of filter paper use during staining for smear microscopy. Yet there are published papers, standard operating procedures (SOP), and commercial kits reporting its use ^8, 10– 13^, without any explanation as to why this step is included in the staining protocol. Therefore, our current study appears to be the first to formally evaluate the role of filter paper during staining in smear microscopy for TB.

It is important to clarify that various types of filters, usually polycarbonate filters (but not standard filter paper) have been used to try to concentrate TB from sputum prior to the polycarbonate filter (instead of the sputum) being used for TB diagnostic testing ^13, 18, 19^. It should be noted that our current research did not assess this experimental use of polycarbonate filters attempting to concentrate TB from specimens, but rather evaluated the application of filter paper to sputum smears only during the staining procedure.

We did not find any significant difference between the number of precipitates visualised in the smears stained with or without filter paper. Furthermore, there were no false positive smears from healthy control samples, with or without filter paper use. Thus, we found no evidence that filter paper removed precipitates from the primary dyes, affecting reading and risk of false positive results. However, it should be noted that during the reagent preparation in our laboratory (and we believe in most laboratories), carbol fuchsin and auramine (but not FDA) were filtered into the bottles used for storing the stains. 

Despite heat fixation of smears, parts of the smear washing away from the slide during the staining process was a significant problem that could potentially leading to false negative results, especially in samples with low bacillary load stained with FDA. We found no overall effect of filter paper on the probability of smear washing away during the staining process. Investigation into methods to prevent smear removal during staining warrants further research. Adding bovine serum albumin prior to heat fixation with or without the use of added ultraviolet light exposure for further cross-linkage for cell adhesion, the use of poly-L-lysine covered slides, or methanol fixation may reduce washing away of smears but increase the costs and/or the time required for smear microscopy ^20, 21^

A strength of this study is its powerful design and analysis, since repeated measurements of each sample under multiple experimental conditions controlled for between-sample variability that would have occurred had we used different samples for each test. This approach generated clear, statistically significant results without risking confusion that could be caused by random variation between samples. Similar to clinical TB laboratories, we did not homogenise the sample before preparing smears, which could contribute to within-sample variability, because *M. tuberculosis* is known to clump within sputum ^22^. To counterbalance this limitation, all slides were read in a standardised manner visualising 100 high-power fields. Another limitation of this study is that despite agreement between investigators and microscopists on what was to be considered a precipitate prior to study commencement, during slide reading there was difficulty in correctly and consistently identifying precipitates.

In conclusion, the results from this study demonstrate that filter paper use during staining for sputum smear microscopy either with ZN, auramine or FDA provided no detectable benefit and reduced microscopy sensitivity. We conclude that filter paper should not be applied to sputum smear during staining in TB smear microscopy procedures.

## Data Availability

Harvard Dataverse: A controlled evaluation of filter paper use during staining of sputum smears for tuberculosis microscopy, https://doi.org/10.7910/DVN/GZQNPW ^23^ Data are available under the terms of the Creative Commons Zero “No rights reserved” data waiver (CC0 1.0 Public domain dedication).
