# Supplementary material for: A controlled evaluation of filter paper use during staining of sputum smears for tuberculosis microscopy
Source: Wellcome Open Res. 2023 Apr 14;8:171. [Version 1] doi: 10.12688/wellcomeopenres.18827.1 (PMC10521092; doi:10.12688/wellcomeopenres.18827.1)
Supplement: Peer review history

# Reviewer report: Reviewer response for version 1

**DOI:** 10.21956/wellcomeopenres.20873.r60216

**Published:** 2023-09-11

**Authors:** Kassu Desta

**Affiliations:** 1 Addis Ababa University, Addis Ababa, Addis Ababa, Ethiopia

The researchers brought important ideas that could improve TB microscopy.

 I have a few comments:

- It is good to indicate the number of new cases and patients on treatment in line with the current work.
- The sample size still is small to discourage the use of filter paper during staining; rather duplication of the work is important in actual TB diagnostic facilities.

This work could be more important also in resource limiting settings with high TB burden.

Is the work clearly and accurately presented and does it cite the current literature?

Yes

If applicable, is the statistical analysis and its interpretation appropriate?

Partly

Are all the source data underlying the results available to ensure full reproducibility?

Yes

Is the study design appropriate and is the work technically sound?

Yes

Are the conclusions drawn adequately supported by the results?

Partly

Are sufficient details of methods and analysis provided to allow replication by others?

Partly

Reviewer Expertise:

I am a Medical Laboratory technologist, specializing in Medical Microbiology.

I confirm that I have read this submission and believe that I have an appropriate level of expertise to confirm that it is of an acceptable scientific standard.

# Reviewer report: Reviewer response for version 1

**DOI:** 10.21956/wellcomeopenres.20873.r65085

**Published:** 2023-08-24

**Authors:** Liya Wassie

**Affiliations:** 1 Armauer Hansen Research Institute, Addis Ababa, Addis Ababa, Ethiopia

The paper reads clear and gives added information in the betterment of *Mtb* microscopic diagnoses. I have a minor comment as described below: Looking at both the absolute #s and proportions on Table 2, the reported difference in percent positivity between no-filtered and filtered staining results seems not statistically different, except for ZN staining (which also looks borderline); this also looks apparent from the overlapping error bars between the two groups shown on Figure 3 and 6; despite this, it is not clear how the P-value became below 0.05. I suggest a statistician to review these sections and comment.   

 I approve the manuscript with this minor consideration.

Is the work clearly and accurately presented and does it cite the current literature?

Yes

If applicable, is the statistical analysis and its interpretation appropriate?

I cannot comment. A qualified statistician is required.

Are all the source data underlying the results available to ensure full reproducibility?

Yes

Is the study design appropriate and is the work technically sound?

Yes

Are the conclusions drawn adequately supported by the results?

Yes

Are sufficient details of methods and analysis provided to allow replication by others?

Yes

Reviewer Expertise:

Immunology, particularly studying host immunity-Mtb interaction

I confirm that I have read this submission and believe that I have an appropriate level of expertise to confirm that it is of an acceptable scientific standard.

# Reviewer report: Reviewer response for version 1

**DOI:** 10.21956/wellcomeopenres.20873.r60209

**Published:** 2023-08-22

**Authors:** Anil Pooran

**Affiliations:** 1 Medicine, University of Cape Town, Rondebosch, Western Cape, South Africa

The authors present a study to assess the effect of using filter paper on the test performance of TB sputum smear microscopy using 3 different staining techniques. The authors suggest that the filter paper may improve dye fixation and reduction of stain precipitates, thereby improving the quality and ‘cleanliness’ of the stain. However, the filter paper reduced smear positivity in terms of lower lower smear grades and AFB counts compared to no filter paper and regardless of the stain used. Furthermore filter paper had no effect of reducing stain precipitates or washing away of the smear during staining. Overall, there was no benefit for using filter paper and is not recommended by the authors.

 Overall, experiments are appropriately designed, well structured and follows a logical pattern, the writing is succinct and the conclusions clearly support the results. The biggest potential pitfall was the use of different sputum samples or even serial samples from the same patient to test the different techniques but the authors have mitigated this well. Overall, it is a well presented study. My biggest concern is the usefulness of the study given there is very limited literature recommending the use of filter paper for smear microscopy.

 My comments are:

1. Being in TB research for a long time, I have never come across the use of filter paper to improve the quality of smear microscopy preps. The authors provide some outdated and rather limited evidence where the use of filter paper is recommended. The authors should give a few more references that are a bit more relevant to support and justify this strategy.
2. It would be interesting to see if the sputum consistency had an effect on positivity and could perhaps be included in the logistic regression analysis (samples are split evenly between salivary and purulent).
3. The result for the washing away of the smear seems very subjective. Furthermore, the filter was removed after the primary stain was applied so the majority of washing away could have occurred in the subsequent staining steps (adding distilled water and counterstain). Not convinced on the usefulness of this result. This should at least be addressed in the discussion.

Is the work clearly and accurately presented and does it cite the current literature?

Partly

If applicable, is the statistical analysis and its interpretation appropriate?

Yes

Are all the source data underlying the results available to ensure full reproducibility?

Yes

Is the study design appropriate and is the work technically sound?

Yes

Are the conclusions drawn adequately supported by the results?

Yes

Are sufficient details of methods and analysis provided to allow replication by others?

Yes

Reviewer Expertise:

Tuberculosis clinical research, immunopathogenesis

I confirm that I have read this submission and believe that I have an appropriate level of expertise to confirm that it is of an acceptable scientific standard, however I have significant reservations, as outlined above.
